# Adapting group care to the postpartum period using a human-centered design approach in Malawi

**DOI:** 10.1186/s12913-023-10036-2

**Published:** 2023-10-14

**Authors:** Ashley Gresh, Anne Batchelder, Nancy Glass, Janet Mambulasa, Esnath Kapito, Amy MacDonald, Nellie Ngutwa, Cori Plesko, Ellen Chirwa, Crystal L. Patil

**Affiliations:** 1 Johns Hopkins University School of Nursing, 525 North Wolfe Street, Baltimore, MD 21231 USA; 2 Kamuzu University of Health Sciences, Private Bag 360 Chichiri, Blantyre 3 Malawi; 3 Pomelo Care, Hillsborough, North Carolina USA; 4 Group Care Global, Philadelphia, PA USA; 5 School of Nursing, University of Michigan, 400 N. Ingalls, Suite 3320, Ann Arbor, MI 48109 USA

**Keywords:** Maternal child health service, Postpartum, Infant health, Preventive health services

## Abstract

**Background:**

Responsive and resilient strategies to reduce high rates of maternal and infant mortality and clinician shortages are needed in low- and middle-income countries (LMICs). Malawi has some of the highest maternal and infant mortality rates globally. Group healthcare is a service delivery model that integrates these strategies. Although primarily implemented during the prenatal period, its potential for improving both maternal and infant health outcomes during the postpartum period has not been realized. The purpose of this study was to adapt and co-design the prototype for an evidence-based group care model for the postpartum period using a human-centered design approach with key stakeholders in Malawi.

**Methods:**

We completed steps of a framework guiding the use of human-centered design: 1) define the problem and assemble a team; 2) gather information through evidence and inspiration; 3) synthesize; and 4) intervention design: guiding principles and ideation. Qualitative methods were used to complete steps 2–4. In-depth interviews (*n* = 24), and incubator sessions (*n* = 6) that employed free listing, pile sorting and ranking were completed with key stakeholders. Data analysis consisted of content analysis of interviews and framework analysis for incubator sessions to produce the integrated group postpartum and well-child care model prototype. The fifth step is detailed in a separate paper.

**Results:**

All stakeholders reported a desire to participate in and offer group care in the postpartum period. Stakeholders worked collaboratively to co-create the prototype that included a curriculum of health promotion topics and interactive activities and the service delivery structure. Health promotion topic priorities were hygiene, breastfeeding, family planning, nutrition, and mental health. The recommended schedule included 6 sessions corresponding with the child vaccination schedule over the 12-month postpartum period.

**Conclusions:**

Using a human-centered design approach to adapt an evidence-based group care model in an LMIC, specifically Malawi, is feasible and acceptable to key stakeholders and resulted in a prototype curriculum and practical strategies for clinic implementation.

## Background

High rates of maternal and infant morbidity and mortality persist in the postpartum period in low- and middle-income countries (LMICs). Globally, sub-Saharan Africa has some of the highest maternal mortality (MMR), severe maternal morbidity, and infant mortality rates [1, 2]. Malawi, a country in the Southern Region has an MMR of 439 maternal deaths per 100,000 live births and for every 1,000 births 42 infants die [1]. While rates of maternal morbidities are unknown in Malawi, in sub-Saharan Africa, rates are estimated as high as 198 per 1,000 live births [2]. Women who experience severe maternal morbidity have been shown to have lower quality of life postpartum, increased risk of mortality, and increased risk of complications including from uncontrolled hypertension, cardiomyopathy, or congestive heart failure [3, 4]. Other morbidities such as postpartum depression, urinary incontinence, obstetric fistula, and sexual dysfunction although perceived as less severe, can be greatly distressing to women [5–7]. Growing data on non-communicable diseases (NCDs) shows that NCDs and maternal health outcomes are inextricably linked, thus contributing to a high proportion of maternal morbidities and mortality beyond the first six weeks after birth [2]. Women may additionally face intimate partner violence (IPV) and sexual and economic coercion in the extended postpartum period, which leads to significantly increased socioemotional problems for both women and infants [8, 9]. The postpartum period, defined for this study as the period from the delivery of the infant up to 12 months after delivery allows for the variation in women’s physical and psychological changes [10] and is an important window for prevention and response across the maternal and child health continuum setting the stage for long-term health, but is often neglected [11]. The first year of life for children is equally crucial for their long-term health and development and maternal morbidities have long-term health consequences for children [12–14]. When responsive caregiving, attachment, cognitive stimulation, and social support are missing, children are more likely to experience negative behavioral, cognitive, social, and emotional outcomes [13–16]. A reimagined form of integrated postpartum and well-child care that sets the stage for long-term health outcomes and well-being for both the mother and her child is needed.

Currently, the World Health Organization (WHO) recommends that every mother and infant have at least four postpartum visits within the first six weeks regardless of birth setting with recommended timing of visits after birth being 1) within 24 h, 2) between 48 and 72 h, 3) between seven and 14 days, and 4) at six weeks [17]. However, although guidelines exist for well-child care, no guidelines exist beyond the first six weeks after birth for women. In Malawi, despite current efforts to improve maternal and infant health and national policies following WHO’s recommended guidelines for postpartum care, rates of maternal and infant morbidity and mortality remain high. Only 42% of women and 60% of newborns receive a postpartum check in the first 2 days after birth and many are not checked or monitored at all in the postpartum period [1, 18, 19]. And only 48% of women are seen by a skilled health worker in the six weeks after delivery [20]. A study examining quality of life in the postpartum period in Malawi found women had lower scores in the domain of physical health than other LMICs [21]. Lack of and poor-quality postpartum care amplifies the vulnerabilities that women and infants in low resource settings face [22]. Responsive and resilient health system strategies are needed to fill a gap in this non-acute period and address preventable maternal and infant morbidities and deaths.

Group healthcare is a health system innovation that can fill this void. Group antenatal (ANC) care has a large body of rigorous evidence supporting its effectiveness and the feasibility of bringing it to scale. CenteringParenting is an emerging model of group postpartum/well-child care that shows promise in advancing guidelines for quality postpartum and well-child care services, addressing gaps in care as indicated by a growing evidence base [23–36]. The core components of this model are healthcare in a group space, interactive learning, and community building [37]. In this model, the same group of 6–8 women and their similarly aged infants attend care together for up to two years [23]. Each group visit is 120 min with the first 30–45 min consisting of self-care (measuring their own infant’s weight and length) and standard health assessments of the infant and mother by a clinician in a separate section of the room. Parent and/or infant referrals are made if needed. This is followed by 75–90 min of interactive learning which includes health promotion, skills building, and support activities (e.g., role plays and facilitated discussions). Group-based care with longer visits provides time and resources to support and address maternal and infant health such as breastfeeding, family planning, depression, nutrition, child development and infection prevention, and by doing this improves quality of care and has the potential to reduce maternal and infant morbidity and mortality [23, 28, 38, 39]. Not only do patients experience positive outcomes, studies show that clinicians also prefer delivering prenatal and postpartum care in a group, due to feelings of increased freedom of expression, and a dissolution of hierarchies [25, 31]. This shift in power dynamics leads to increased quality of care, which can ultimately improve outcomes [25, 31].

Centering-based group care models have not been widely implemented in the postpartum period in LMICs. A group care model provides an opportunity to deliver integrated postpartum and well-child care to childbearing parents and infants beyond the first 6 weeks after birth filling a gap in the care continuum. This has the potential to address physical, mental, and health related social needs that persist beyond the first six weeks postpartum such as postpartum depression and non-communicable diseases. When implemented in antenatal are, group care models are acceptable, feasible, and have improved outcomes including health literacy, prenatal and postpartum attendance, the number of health facility births, and breastfeeding practices [40–45]. Expanding group care into the postpartum period offers a promising strategy to reduce gaps in the care continuum. Therefore the purpose of this study was to use qualitative methods to adapt and co-design the prototype for an evidence-based group care model for the postpartum period using a human-centered design approach with key stakeholders in Malawi.

**Fig. 1:**
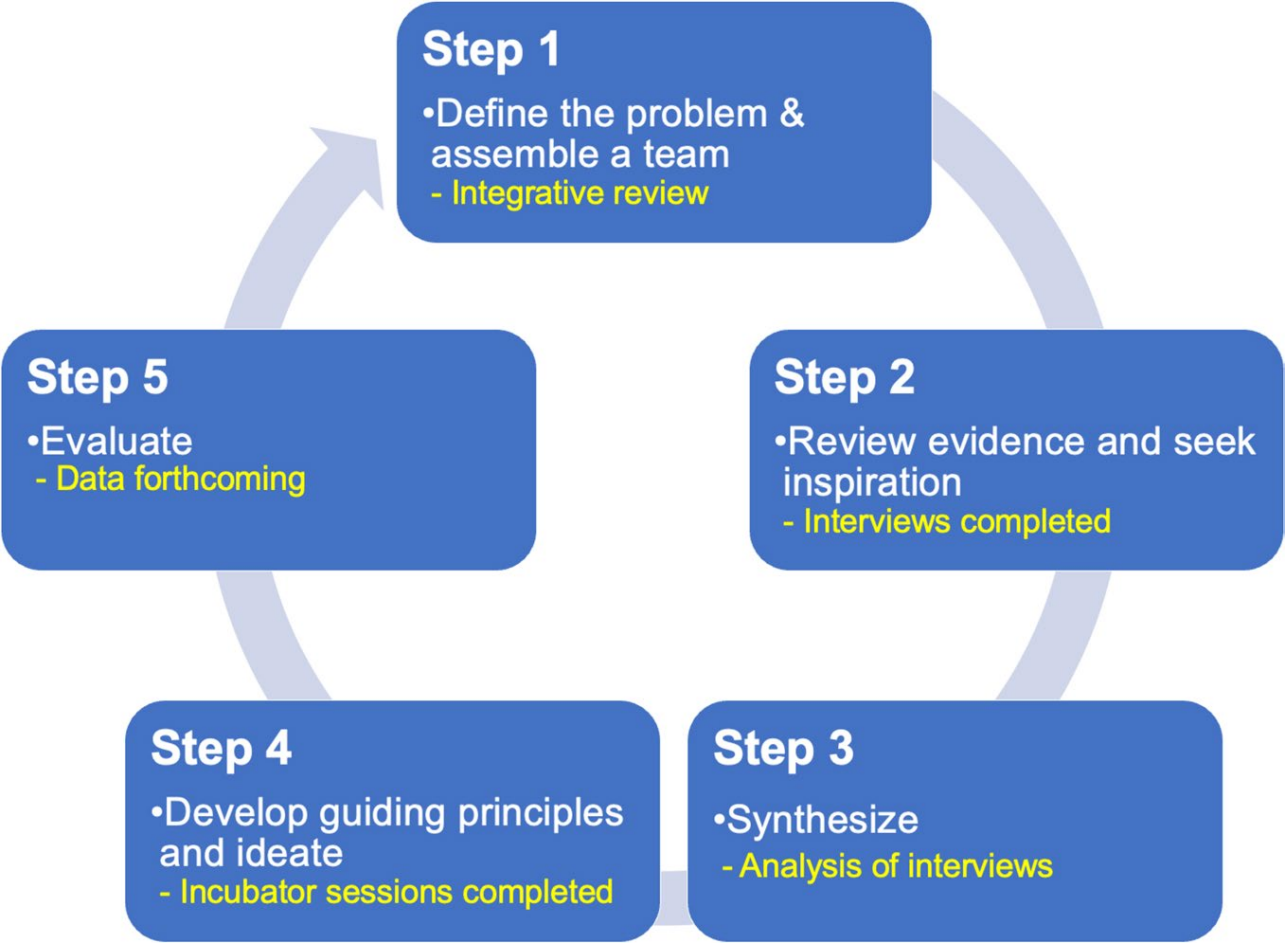
AHEAD Framework for rigorous human centered design to produce the group postpartum/well child prototype

Human-centered design (HCD) offers an approach to co-design and safely, efficiently, and effectively adapt evidence-based interventions such as Centering-based group healthcare to new contexts. HCD emphasizes the strengths, agency, and priorities of women and health care workers to build a model of care that is resilient and responsive to individual, and system needs. This approach reframes a research question or behavior change from “what matters” to “what matters most,” allowing for solutions that are human centered and context specific [46, 47]. Previous healthcare research supports the use of this approach, as participating in the co-design process increases self-efficacy for both patients and health care workers and leads to sustainable solutions to problems within the health system [48–55]. While HCD is an increasingly used approach to finding healthcare solutions, recent systematic reviews of HCD in healthcare have found discrepancies in the quality and methodological rigor of the studies [56, 57]. The Approach to Human-Centered, Evidence-Driven Adaptive Design (AHEAD) framework provides a practical guide to co-design solutions to healthcare challenges [47]. The five steps of AHEAD framework (Fig. 1) include: 1) define the problem and assemble a team; 2) review evidence and seek inspiration; 3) synthesize; 4) develop guiding principles and ideate; and 5) evaluate [47]. This paper seeks to showcase how to use HCD with methodological rigor and the steps to adapting and implementing an integrated group postpartum and well-child care model that is context specific and responsive to people’s needs and desires.

## Methods

### Study design and setting

This formative qualitative study was implemented in three primary care government health centers in Blantyre District, Malawi. These three health centers participated in a trial, “Group Antenatal Care: Effectiveness for Maternal/Infant and HIV Prevention Outcomes and Contextual Factors Linked to Implementation Success in Malawi,” (ANC Trial, UIC IRB #00403255), and have been implementing and sustaining group antenatal care (ANC) since 2019 [58]. The government-run health centers were explicitly selected to represent a range of variation in size and staffing with urban, peri urban, and rural representation. This sample of health centers was also chosen to assess whether government-run health centers can implement group care under everyday conditions without major resource shifts [58]. Key stakeholders included health workers and patients familiar with group healthcare who are poised to provide in-depth perspectives on the benefits and challenges of group-based care. Experienced group ANC facilitators, midwives and community volunteers, and multiparous patients who completed group ANC and are receiving maternal and child health care were asked to participate. Each stakeholder provides valuable insight to inform the design of the integrated postpartum and well-child care group care prototype.

### Sample and recruitment

#### Patients

Over the two months of this study, purposive sampling was used to recruit 12 women. Eligibility included participation in the group ANC trial and having two or more biological children. The rationale for multiparous women was to allow for comparisons across multiple pregnancies and experiences with postpartum and well-child care services.

#### Health care workers

Health care workers included midwives, community volunteers, and health surveillance assistants (HSAs) who were approached to participate. HSAs are a type of community health care worker paid by the government to serve as a link between communities and the health care sector, they have secondary school education, and receive 12 weeks of training and provide many of the under-five health care services, such as vaccinations and growth monitoring [59]. Convenience sampling was used to recruit health care workers since there are a limited number of health care workers with group healthcare experience and that are working in postpartum and well-child care at each of the three clinics. Eligibility criteria for health care workers included the ability to read/speak Chichewa and/or English at a grade 8 level and that they had been working in postpartum or well-child care for at least one year. Exclusion criteria for the study included marked cognitive impairment that would prevent providing informed consent and if they did not speak and understand Chichewa (the national language). The rationale for recruiting health care workers was to provide input and insight to adapt and co-design the integrated group postpartum and well-child care model from the perspective of those that deliver postpartum and well-child care.

### Ethics

This study was approved by the Institutional Review Boards at Johns Hopkins University School of Nursing (IRB #00245018), Kamuzu University of Health Sciences (IRB #P.06/21/3341), and the University of Illinois Chicago (IRB #2022–0327). The study team also obtained an endorsement from the Malawian Ministry of Health’s Blantyre District Health Office to conduct this work.

## Procedures

The five steps of the AHEAD framework are summarized in Fig. 1. Given the iterative nature of HCD, we detail the procedures of the qualitative methods used for each AHEAD step along with the analysis and results since each step informs the next.

### Step 1: define the problem and assemble a team

To define the problem, in-depth exploration of people’s needs to identify a problem needing a solution is necessary [47]. Once the problem is defined then it is recommended to assemble an interdisciplinary team [47]. To define the problem, we completed an integrative review [60] to describe and evaluate current postpartum care content and service-delivery models used throughout the African continent. Guided by the WHO’s Maternal Morbidity Working Group’s conceptual framework for healthcare interventions to address maternal morbidity, we also identified multiple gaps in care. We showed that NCDs, intimate partner violence (IPV) screening, mental health, and a rights-based approach to care are rarely included [60]. We found that in Malawi specifically there were high rates of maternal mortality and morbidities and poor quality or absent postnatal care [18, 19]. Group care and integrated maternal and child health services models were identified as ways to improve maternal and child health outcomes [60]. We concluded that a standardized package of postpartum care that can be adapted for specific contexts from birth to one year postpartum is needed to further reduce maternal and infant morbidities and mortality [60].

The results of this review supported moving to Step 2. We leveraged existing partnerships, research infrastructure, expertise, and momentum from the ANC trial to assemble an interdisciplinary team to guide the adaptation and development of an integrated group postpartum and well-child care model.

### Step 2: gather information through evidence and inspiration

This step involves “information gathering to identify themes for potential solutions” [47]. In-depth interviews [61] were conducted with key stakeholders to inform the design of group postpartum and well-child care. Interview guides were developed using open-ended questions to explore women and health care worker’s experiences of postpartum and well-child care, identify priority health promotion topics to include in the prototype and explore perspectives on extending group care into the postpartum period. Interview guides were first translated from English to Chichewa by a committee consisting of researchers, a bilingual midwife, and two lay people [62]. The interview guides were piloted and refined to ensure appropriate framing and sequencing. Following an informed consent process, written consent was obtained, and interviews were conducted in Chichewa by a trained research assistant who is also a midwife in a private room reserved at the clinic and lasted approximately 45 min. The intent of the format and interview questions was to encourage participants to talk openly and solicit rich accounts about current postpartum and well-child care practices, patient flow, equipment/supplies, culturally appropriate services, postpartum health concerns, desired health promotion topics, and perspectives on group healthcare. For example, women were asked, *what were your health needs during the postpartum period?* Health care workers were asked, *what topics do you think are important to cover in creating content for postpartum visits?* We achieved thematic saturation after completing twenty-four audio-recorded interviews with 12 women, 4 midwives, 4 community volunteers, and 4 HSAs across the three clinics. Audio-recordings were transcribed and translated by the research assistant who conducted the interviews. To ensure translation accuracy, translations were reviewed by a team consisting of researchers, a bilingual midwife and two lay people so that English transcripts would be ready for the Step 3 analysis. To promote rigor and trustworthiness, the procedures adhered to recommended qualitative research guidelines and the consolidated criteria for reporting qualitative research (COREQ) [63, 64].

### Step 3: synthesis of qualitative data from step 2

This step involves identifying emerging themes from the information gathering [47]. To complete this step, transcribed interviews were stored, managed, and analyzed using Dedoose software. A content analysis approach was used to analyze interviews, and a category system was created based on the health promotion topics covered in the original Centering-based group care model for coding themes using the interviews as the unit of analysis [65]. New codes were added as needed. Two team members coded each transcript separately and then met to compare and finalize the codebook. Although the analysis was largely deductive, we allowed for the inclusion of emergent codes, as we know that all health promotion topics and content areas of the original model may not be comprehensive of experiences in Malawi.

Women ranged in age from 19 to 41 years and had between 2 and 4 children. Health care workers were 35 to 58 years old and their years of experience in their current role ranged from 1 to 21 years. The information and insights gained through this analysis provided an empathetic view of what is currently offered as part of postpartum and well-child care in the Malawian context and allowed the women and health care workers to express what mattered most to them as they identified postpartum and well-child care service and health promotion priorities.

We identified five themes when analyzing both the health care worker and mother’s responses: 1) maternal health assessments are not consistently completed; 2) challenges exist to postpartum and well-child care attendance and delivery of care; 3) postpartum and well-child health promotion topics are not standardized; 4) maternal and child health concerns included physical and psychological issues; and 5) there is buy-in for the group healthcare model from both women and health care workers. See Table 1 for illustrative quotes by theme.

#### 1) Maternal health assessments are not consistently completed

When women and health care workers described the current one- and six-week postnatal visits they reported that visits often focused on the infant health assessment, “*At 6 weeks they [women] don’t meet us; they go for vaccines*.” Most women did not get a physical assessment although midwives did describe discussing danger signs at the postnatal visits. Women were instructed by health care workers to bypass 6-week postnatal check and go straight to the under-five clinic. A woman noted that she was too afraid to ask questions or ask for services because of the negative attitudes of health care workers. While the health care workers recognized the need for thorough physical examinations of the woman and infant and wanted to complete them, they often cited staff shortages and lack of equipment as reasons for incomplete exams.

#### 2) Challenges exist to postpartum and well-child care attendance and delivery of care

Participants identified individual, structural, economic, and environmental factors related to attendance and delivery of services (see Table 1 for illustrative quotes describing these challenges). In addition to describing the clinic-level challenges, some midwives took personal responsibility for the lack of postnatal care, “*I think the gaps are many, but I think for most of the gaps, we are the ones that create them*” Health care workers also explained that the rainy season, difficult terrain, and long travel distances made getting to the clinic a challenge for their clients. Others cited poverty and relationship conflicts as additional access barriers for women.

#### 3) Postpartum and well-child health promotion topics are not standardized

**Fig. 2:**
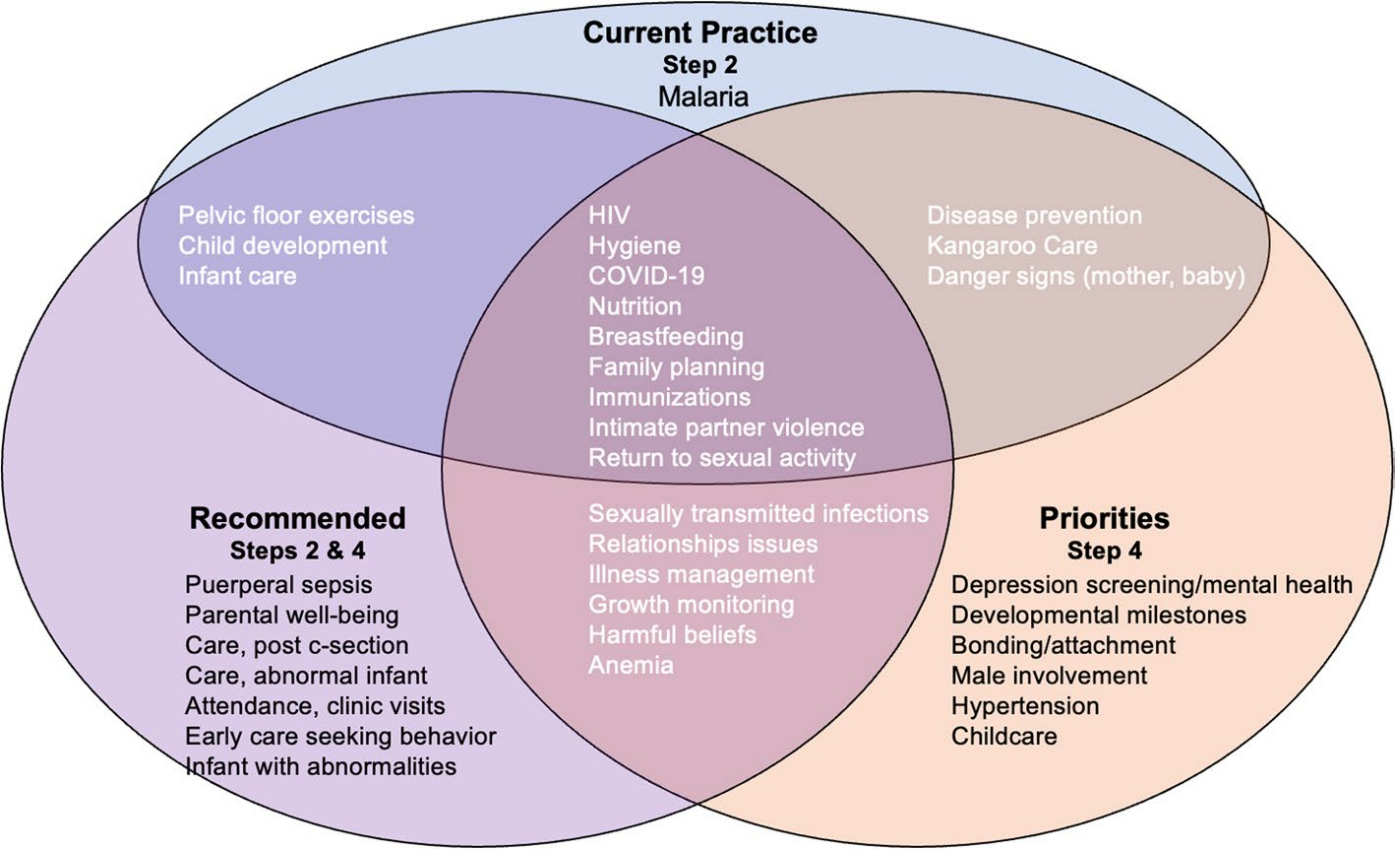
Health promotion topics described as current practice, recommendations, and/or priorities

When asked to identify which health promotion topics were currently discussed at clinic, responses varied and were inconsistent (see Fig. 2, topics in the blue circle). Some mothers expressed that they did not receive any health education, “*At the moment, they don’t give any health education”.*

#### 4) Maternal and child health concerns included physical and psychological issues

When asked what common maternal health concerns they experienced responses included: difficulty breastfeeding, poor nutrition, IPV, sexually transmitted infections, mental health, high blood pressure, malaria, complications post c-section, late in seeking care for health issues, and sepsis. When asked about common child health concerns they described: cord infections, malnutrition, poor growth, eye infections, jaundice, skin problems, diarrhea, pneumonia, and coughs and fevers.

#### 5) There is buy-in for the group healthcare model from both women and health care workers  (Table 1)

**Table 1:** Themes from interviews with participants and illustrative quotations

Theme	Illustrative Quotations	
	**Health Care Workers**	**Women**
**Maternal health assessments are not consistently completed**		
1-week visit	• At the first week, we are assessing if the baby is breastfeeding well; we ask them if they have experienced any problems. So, if they say there is no problem then we do an assessment of the baby. Midwife 2, Clinic B	• They weighed the baby, then she was 2.5 kgs but his birth weight was 2.9 kgs. And then they said that I should go and get vaccine for the baby; they gave the baby an injection on the arm and then also some on the tongue…they did not ask me how I am doing…They checked the baby on the cord, and they also gave me iron tablets. Mother, Clinic C
6-week visit	• At 6 weeks they [women] don’t meet us; they go for vaccines. Midwife, Clinic A	• [At 6 weeks] I reported here…they checked the baby on the cord and then they told me to go for immunization. Mother, Clinic C
	• [At 6 weeks] they report at the under-five clinic but if they have problems, they come back to the maternity. But those who are ok don’t come back unless if the woman wants family planning methods. Midwife 2, Clinic A	
**Challenges exist to postpartum and well-child care attendance and delivery of care**		
Responsibility of health care workers and their attitude	• I think the gaps are many, but I think for most of the gaps, we are the ones that create them; may be because sometimes we have work overload, and we tend to skip most of the things that are supposed to be done with the woman. But we have everything that we need to tell the women…So it all starts with us; we need to teach them. At one week they come but at 6 weeks to be honest they don’t come to be reviewed by the nurse, they just come for a vaccine, if they come here, it’s because they have a problem. Midwife 2, Clinic A	• At the moment, they [health care workers] don’t give any health education. Mother, Clinic C
	• …If the health workers’ attitude is good, women come to the under-five clinic, but if the attitude of the health worker is not right, women stop coming to the clinic. They seek care from another place. HSA 2, Clinic B	• We know the baby is supposed to get vitamins every 6 months. But you find that 6 months have elapsed, and the baby is not given the vitamins. So sometimes we just look, we can’t ask because we are afraid. Mother, Clinic B
Lack of resources (e.g., staff shortage, lack of equipment)	• But sometimes we are busy, when the woman just says that she is fine, you just continue without paying attention to the woman, not knowing that she has other issues. But because she didn’t say, you don’t know and because you didn’t inquire, then the woman is not properly assisted. Midwife 2, Clinic B	• At the under-five clinic, the care is not that much; I am saying that because like today, they have weighed the baby; and the people that weighed the baby are strangers. Mother 3, Clinic B
	• We need to also be checking vital signs but at this facility we don’t have equipment. Midwife 1, Clinic A	
Perceived lack of knowledge	• We can also say it is a lack of understanding on the importance of postnatal care. So, to them, it’s enough if the baby got the initial vaccine. The rest is not important to them; I also think it’s because of lack of knowledge. So, we just need to sit down with them and explain in detail, so they understand. Midwife 1, Clinic C	• Another thing that I noted was that the first vaccine that she was supposed to receive that day she did not receive. I don’t know maybe they took it that we work here, and we know these things. But to me I didn’t know. So, the baby was not given BCG…. They should have made sure that before I was discharged, the baby has received all the vaccines that she was supposed to receive. My baby didn’t receive any. What I would have loved was that the nurses should have make sure that we have received everything that we were supposed to receive. Mother 1 Clinic B
Environmental	• …there are some who are very far, but we fail to reach out to them because of floods. You find that a place which is close by, become inaccessible during rainy season… HSA 1, Clinic C	
	• The issue of distance in this area, being a hilly area, and also rivers during rainy season, make women not come to the clinic if it’s raining…The distances are very long and it’s difficult for a woman to travel with a small baby. Midwife 2 Clinic B	
**Maternal and child health concerns included physical and psychological issues**		
Physical	• Then another problem is nutrition; when they tell you that they baby is not having enough breastmilk, you can see that the nutrition of the mother is very poor. And you can see that the way the woman is looking she is poor. Midwife 1, Clinic C	• The other problem was that I was having problems to walk. It was because of the tear; I was even having problems to carry the baby so what happened was that I could lay down. Mother, Clinic A
	• Malnutrition is about 10–12% of all the children that we see at the under-five clinic. HSA 2, Clinic C	• But sometimes the problem is with the child, difficult to eat and you find that the weigh is not increasing yet the food is there. Mother, Clinic B
	• But most of the women when they come, we find that their BPs are very high, so we give them medication. Midwife 1, Clinic A	• They were giving me the blood pressure medication…They told me that the I should be taking the medication and then after 7 days I should stop. Mother, Clinic B
	• Most of the times the women don’t say, but sometimes they would tell you that the baby had diarrhea. For the first 6 months we have common problems like diarrhea and also malaria…But we emphasize a lot on the growth monitoring and if we see that the weight has dropped a lot, we refer women to the nutrition unit. HSA 1, Clinic C	
Mental health	• On the psychological, we check so many things; like here, most of the women have children with men that left them, and they don’t have any support for the child; some women are staying with someone who abuses them, and this affects how they are breastfeeding the baby. Midwife 2, Clinic A	• They also told me that when a woman has just delivered, sometimes you may have psychological problems so when I feel like that, I should rush to the hospital because it shows that something is happening in the body. Mother 3, Clinic B
**Health promotion topics discussed in both postpartum and well-child visits are not standardized**		
	• See Fig. 2 for list of health topics currently being discussed at health visits	
**There is buy-in for the group healthcare model from both women and health care workers**		
	• I believe that the group postnatal care could assist a lot because there are so many things that the women don’t know; and when they are in groups and they receive counseling, I feel this can help to improve their well-being, both the mother and the baby. Because I strongly feel that these women can take very good care of their babies, they only need enough time to get enough counseling. But I feel that if you can adopt that model, it will be very helpful. HSA 1, Clinic C	• I think that [extending group care into the postpartum period] would be a very good thing because if like the way we were doing when we were pregnant, we were sharing ideas so that we should not be in the dark, so I think that if we do the same thing now that we have delivered, it can still help us; we can still share ideas. For example, if someone has a problem and she shares it on the group, we can help each other. For example, the way I was struggling with my baby when she was having fevers, there could be some women who had also experienced that, and they know exactly what to do They could have assisted me. Maybe it’s at night, I can’t come to the hospital right away, those advice help. So, to me, I think the group care approach is the best; it is very helpful. Mother 1, Clinic B
	• That can work very well, and it can be very good. Because as for us we just see the babies once, but that can assist us to be following up on the babies to some point and be monitoring them, so it can be very good because it will bring change. It can also improve the way we do our work because then the babies can also be seen at 6 weeks. So, it is very good so many things can change. Midwife 1, Clinic A	• I would have loved if these groups continued. Sometimes things happen in the village; emergencies and the doctor is not readily available you can assist someone and save a life because at least you know some things. Mother 2, Clinic B

Mothers reported a desire to participate in group care and health care workers reported interest in offering group care during the postpartum period. Mothers felt that the group care model was supportive and helped them translate knowledge into practice and better address health concerns. Health care workers expressed that group care could improve overall quality of care, “*I feel this can help to improve their well-being, both the mother and the baby*.”

### Step 4: intervention design: developing the guiding principles and ideation for content and structure of the integrated group postpartum and well-child care prototype

This step involves creating guiding principles that align with an evidence-based model, and then rounds of brainstorming and prototyping [47]. Building on the Step 3 synthesis, we carried out incubator sessions with key stakeholders. A total of six incubator sessions, two at each clinic, were completed with one to three women and two health care workers, a midwife, community volunteer, or HSA. Incubator sessions are like focus groups, but the emphasis was on developing the health promotion content, interactive learning activities, and structure of the group postpartum/well-child care model through brainstorming, co-creation, and finding solutions. Multiple qualitative methods supported this process including free listing, pile sorting and ranking [66, 67]. Free listing, successfully used in public health research [66], is a method for rapidly gathering information on a topic by listing as many ideas as possible related postpartum and well-child care. This approach allows for examining intracultural variations of postpartum and well-child care and provides opportunities to build consensus about healthcare services priorities [50].

Following an informed consent process, written consent was obtained, and incubator sessions were conducted in Chichewa by a trained research assistant. In-person incubator sessions lasted 1–2 h and took place in a private room reserved at each health center. Participants were asked to free list what they wanted to be addressed in postpartum and well-child care and their responses were recorded on a flip chart.

The research assistant then presented and described each health promotion topic generated from Steps 2 and 3 (see Fig. 2, blue and purple circles) and health promotion topics and activities included in Centering-based group care model to participants. Participants were instructed to confirm or reject each of these. We retained all topics that had a majority vote among participants. Then each item was written on a card which the research assistant read aloud to participants during a group pile sorting and ranking activity [67]. Participants worked collaboratively to rank the topics and generate a prioritized list for the final prototype. Participants were then asked to identify which type of group ANC interactive activities (e.g., games and role plays), should be retained in the final prototype.

Participants were asked to explore the ideal visit length and structure and provide feedback on how to implement group postpartum/well-child care feasibly and sustainably at their clinic. Other implementation factors were asked about including resource availability, scheduling, follow-up care, and ways to integrate this service delivery model within the existing infrastructure.

Incubator session data analysis was iterative and included continuous data integration and consultation with key stakeholders. The set of codes were analyzed using a framework method for qualitative analysis [68] because the defining feature of this method is a matrix output of summarized data. The framework method includes seven analytical stages: 1) transcription and translation of transcripts; 2) familiarization with the interviews/focus groups; 3) coding (An initial set of codes was developed based on the health promotion topics and activities included in the Centering-based group care model; 4) developing a working analytical framework using a few transcripts after initial coding (codes are grouped together based on sessions and content areas); 5) applying the analytical framework to remaining transcripts using existing categories and codes (new codes were added as they emerged); 6) charting data into the framework matrix (in an Excel spreadsheet to manage and summarize data); and 7) interpreting the data [68].

The iterative process of qualitative data collection and analysis from Steps 2–4 were used to ultimately produce the co-designed integrated group postpartum and well-child care prototype which we present in the results section below.

## Results

Through the above qualitative methods and data analysis we co-designed an integrated group postpartum and well-child care prototype specific to the Malawian context. Below we describe the health promotion topics that were identified as priority topic areas to include in the interactive learning component of the model that reflect what women and health care workers reported are most important to their health and health related social needs, the suggested service delivery structure of the model, and a description of the final prototype.

### Health promotion topics

At incubator sessions when the Step 2 current and recommended health promotion topics were presented to participants, they confirmed that all the presented health promotion topics should be included in the group postpartum/well-child care prototype. See Fig. 2 for a comparison of what was described in current practice during the interviews (Step 2), what was recommended during interviews (Step 2), and what health promotion topics were prioritized in Step 4.

During the free listing activity, seven additional health promotion topics were added such as how to care for babies with abnormalities and male involvement. Women and health care workers often provided explanations for why they thought each one was important that reflected their prior experiences. As part of the prioritization process, participants also linked topics that should be introduced together (e.g., family planning and return to sexual activity; Table 2).

**Table 2:** Number of incubator sessions (*n* = 6) that prioritized each health promotion topic

Session						Health Promotion Topic
**1**	**2**	**3**	**4**	**5**	**6**	
▪	▪	▪	▪	▪	▪	Hygiene^a^
▪	▪	▪	▪	▪	▪	Exclusive breastfeeding
▪	▪	▪	▪	▪	▪	Family planning (combined with return to sexual activity)
▪	▪		▪	▪	▪	Nutrition
▪	▪		▪	▪		Depression screening/mental health
	▪		▪	▪	▪	Growth monitoring
		▪	▪	▪	▪	HIV Prevention of Mother-to-Child transmission^a^
▪	▪		▪			Immunizations
	▪	▪	▪			Danger signs for mother and baby
		▪		▪	▪	Intimate partner violence
			▪	▪	▪	Sexually transmitted infections
▪		▪				Anemia/Iron supplements^a^
	▪			▪		Developmental milestones
▪					▪	Illness management
		▪			▪	Disease prevention
		▪		▪		COVID-19^a^
	▪	▪				Kangaroo care^a^
		▪			▪	Harmful cultural practices^a^
▪						Male involvement
▪						Relationship issues
	▪					Childcare
		▪				Hypertension^a^
					▪	Bonding/attachment

^a^Indicates content areas added to the facilitator’s guide that were not formally included in CenteringParenting and are responsive participants’ identified priorities

The final list of health promotion topics included in the prototype were: hygiene, exclusive breastfeeding, family planning (combined with return to sexual activity), nutrition (for both mother and infant), depression screening/mental health, growth monitoring and developmental milestones, sexual health (including STIs and HIV), immunizations, danger signs for mother and baby, IPV and relationship issues, mother’s physical health (including anemia, hypertension, and cervical cancer screening), disease prevention and management (including COVID-19 and common maternal and child health concerns), and male involvement. The preferred interactive learning activities were role plays and discussions.

### Structure of implementation

There was consensus across all three clinics to create a 6-visit model with recruitment occurring at the 1-week postpartum visit and the first group visit beginning with the 6-week postpartum visit. This varies from CenteringParenting’s 9-visit model. Participants desired to maintain individual visits for the recommended Malawian guidelines for postpartum visits at 24 h, 48–72 h, and one week after birth. They suggested that the schedule correspond with the child vaccination schedule (6, 10, and 14-weeks and 6, 9, and 12-months) with each group 1–2-h visit being co-facilitated by a midwife and an HSA. Some hypothesized that this schedule would increase vaccine uptake since vaccines would be administered at the end of each visit. Responses varied for the ideal group size ranging from 8–15 mother/infant dyads.

Participants outlined a set of resources and materials needed to successfully provide high quality care in a group format that included: training, an adult and baby weight scale, blood pressure equipment, thermometer, tape measure, an examination bed, mattress protectors, a mat to sit on, privacy screen for in the room, physical assessment guidelines, hand sanitizer, laminated pictures of danger signs to show women, flip charts, toys for babies, and a storage basket. Many health care workers stated they did not have access to guidelines to perform standardized physical assessments. So, they recommended inclusion of written clinical maternal and infant assessment guidelines for each visit to standardize and improve the quality of these health assessments.

### The prototype

As part of Step 4, a curriculum and implementation structure prototype for group postpartum/well-child care was produced. The prototype consisted of a facilitator’s guide outlining each of the six visits with a detailed plan for each visit that included the associated clinical guidelines for physical assessments and objectives and directions for each interactive learning activity reflecting the prioritized health promotion topics (Table 2), as well as an implementation plan. The facilitator’s guide was adapted from Centering-based group care to 1) include all priority health promotion content areas desired by women and health care workers; 2) be inclusive of activities for low literacy levels; and 3) translated into Chichewa the local language. For example, for the six-week visit, interactive learning activities focus on danger signs for mom and baby, physical and emotional adjustments after having a baby, breastfeeding, and family planning/resuming sexual activity. Each visit outlines all activities necessary to maintain model fidelity to Centering-based group healthcare’s core components of healthcare in a group space, interactive learning, and community building.

Seven midwives experienced in group care (5 from Malawi including the principal investigator of the Group ANC Study, and 2 in charge of clinics participating in this study) and 2 registered nurses (1 specializing in pediatric care) validated the content and structure of the facilitator’s guide and implementation plan. The expert review process happened over a period of several months and involved multiple rounds of review to ensure all content adhered to evidence-based practice for postpartum and well-child care. Based on their input content was added or refined and irrelevant content was removed. The clinical guidelines for assessments for women and infants and developmental milestones infants are expected to achieve at each visit in the facilitator’s guide were reviewed to ensure that these adhered to the guidelines and recommendations of the American Academy of Pediatrics Bright Futures [69], WHO [17] and Malawian Ministry of Health (e.g., the National Reproductive Health Service Delivery Guidelines and the Guidelines for Maternal and Newborn Health Services Including Family Planning During the COVID-19 Pandemic [70]). The finalized facilitator’s guide has been translated into Chichewa and is ready for the final step in the AHEAD framework which includes a pilot test and evaluation.

## Discussion

Our prototype of integrated group postpartum and well-child care was produced using an HCD approach that was guided by the AHEAD framework; it is now ready for rigorous evaluation [47]. The results of the first four steps of the AHEAD framework showed that group care is a promising strategy for improving health outcomes in the postpartum period [60]. The evidence and inspiration from engaging with women and health care workers reinforced findings from other studies that there are gaps in the availability and provision of postpartum care to mothers in Sub-Saharan Africa [71]. The HCD approach lays the foundation for a responsive and resilient adaptation of group care that is co-designed by women, health care workers, and researchers. As with other studies using HCD approaches this leads to sustainable solutions to health care issues [55, 56, 72, 73]. The reciprocity and power sharing that is inherent to the co-design process of the AHEAD framework offers a strategy to reduce medical hierarchies and center the voices of patients receiving care and health care workers providing it [74]. This iterative process laid the foundation for a flexible model that can respond to the changing and dynamic needs of patients and health care systems.

Prioritized health promotion topics included a range of physical, psychological, social, and behavioral issues. Consistent with current literature, there were noticeable differences in topics desired and prioritized compared to current practice, particularly related to mental and sexual health [75, 76]. The acknowledgement of the high prevalence of perinatal mental health disorders is growing globally and there is a call for models of care that integrate mental health into primary care to improve outcomes for women, which is particularly critical in the postpartum period [77]. Interestingly, malaria was not prioritized or recommended through this work which might signal that people feel they have adequate knowledge and understanding of prevention and treatment strategies. Participants noted several social factors and prioritized male involvement in care and relationship issues. These are important to integrate into the model because it is well-established that the social context of patients’ lives has implication for maternal and child health outcomes that need to be addressed clinical practice [78]. The priority placed on both maternal and child nutrition, malnutrition, and anemia in women highlights the many forms of undernutrition persisting among women and children in many LMICs [79]. Group care during the first 1,000 days offers a promising strategy to identify and manage health issues such as undernutrition that is likely to have the highest gains and improve the nutritional status of women and children during this critical period in the life course [79].

### Lessons learned and implications for practice

Our team had three major lessons learned using the AHEAD framework which we believe are important to consider in undertaking a similar process in other contexts which are: 1) using this approach is time intensive; 2) building a strong interdisciplinary team is key to success; and 3) using multiple methods to engage a range of literacy levels is important. It is important to allocate ample time to truly engage in these formative steps to inform model adaptation and development. The interdisciplinary team allowed for a strong foundation to work from, and the multiple perspectives were invaluable to co-creation and adaptation of the model. And finally, during incubator sessions multiple brainstorming methods were needed to meet people at their levels of literacy, which was critical to engaging all involved in this co-creation process. In addition, it is important to note that this process could face additional challenges in clinics that are not familiar with the group care model. In clinics where group care has not been implemented more time would be needed to introduce the concept of group care to stakeholders to then engage in co-creation and adaptation process.

The group postpartum and well-child care prototype and model offers a strategy to reduce maternal and child morbidity and mortality by integrating maternal and child health care and placing a focus on health promotion and prevention activities addressing NCDs, health-related social needs, and factors that affect morbidity beyond the immediate postpartum period within each visit which aligns the WHO’s Maternal Morbidity Working Group’s conceptual framework for healthcare interventions to address maternal morbidity [80]. A shift to a group healthcare model is an innovative strategy for strengthening health systems because a model, informed by the steps of the AHEAD framework has flexibility to be responsive and resilient to changing contexts and provides a strategy to adapt the group postpartum and well-child care model to meet changing needs in the future and to diverse contexts [80]. The interactive learning component of this model increases the total amount of time that each mother receives health promotion in the first year of life. This adult learning approach is responsive to group needs and provides opportunities to exchange ideas as group members work to better understand and ultimately apply lessons learned to their lives. By transforming the delivery modality of postpartum and well-child care to an integrated group healthcare model, the needs of the mother and the infant are met efficiently at the same time. This model seeks to identify and address the underlying causes of maternal and child morbidities and places importance on the health system’s role in influencing health outcomes.

The implementation plan created reflects one that is responsive to the desires of women and the realities of the health care system based on shared decision making during the incubator sessions in dialogue with both patients and health care workers. The next step in the AHEAD framework will be to pilot all 6 sessions included in the prototype with women and their infants in the postpartum period and assess its feasibility, acceptability and appropriateness among women and health care workers. 

## Conclusions

Using a human-centered design approach to adapt this model of care for the Malawian context centers co-creation, collaboration, and coordination at the patient, clinician, and health system levels; this sets the stage for a responsive strategy to fill a critical gap in the care continuum and improve maternal and child health outcomes. This integrated maternal health and well-child group care model may serve as a transformative approach to filling a neglected area of the care continuum that meets the needs of the dyad in the first year postpartum.

## Data Availability

The datasets generated and/or analyzed during the current study are not publicly available to maintain confidentiality of participants but are available from the corresponding author on reasonable request.

## Abbreviations

LMIC: Low- and middle-income country
MMR: Maternal mortality rate
NCD: Non-communicable disease
ANC: Antenatal care
HCD: Human-centered design
AHEAD: Approach to Human-Centered, Evidence-Driven Adaptive Design
WHO: World Health Organization
HSA: Health surveillance assistant
IPV: Intimate partner violence
COREQ: Consolidated Criteria for Reporting Qualitative Research
HIV: Human Immunodeficiency Virus
STI: Sexually Transmitted Infection
COVID-19: Coronavirus disease
